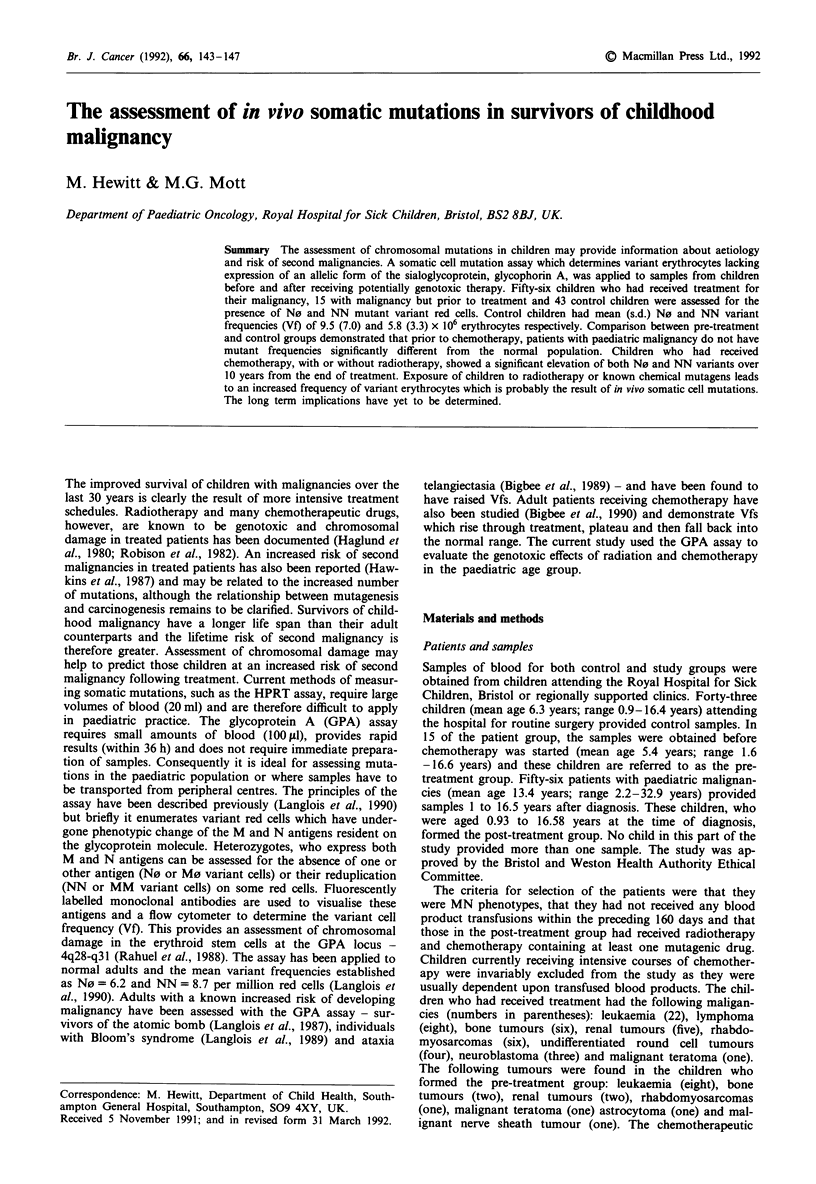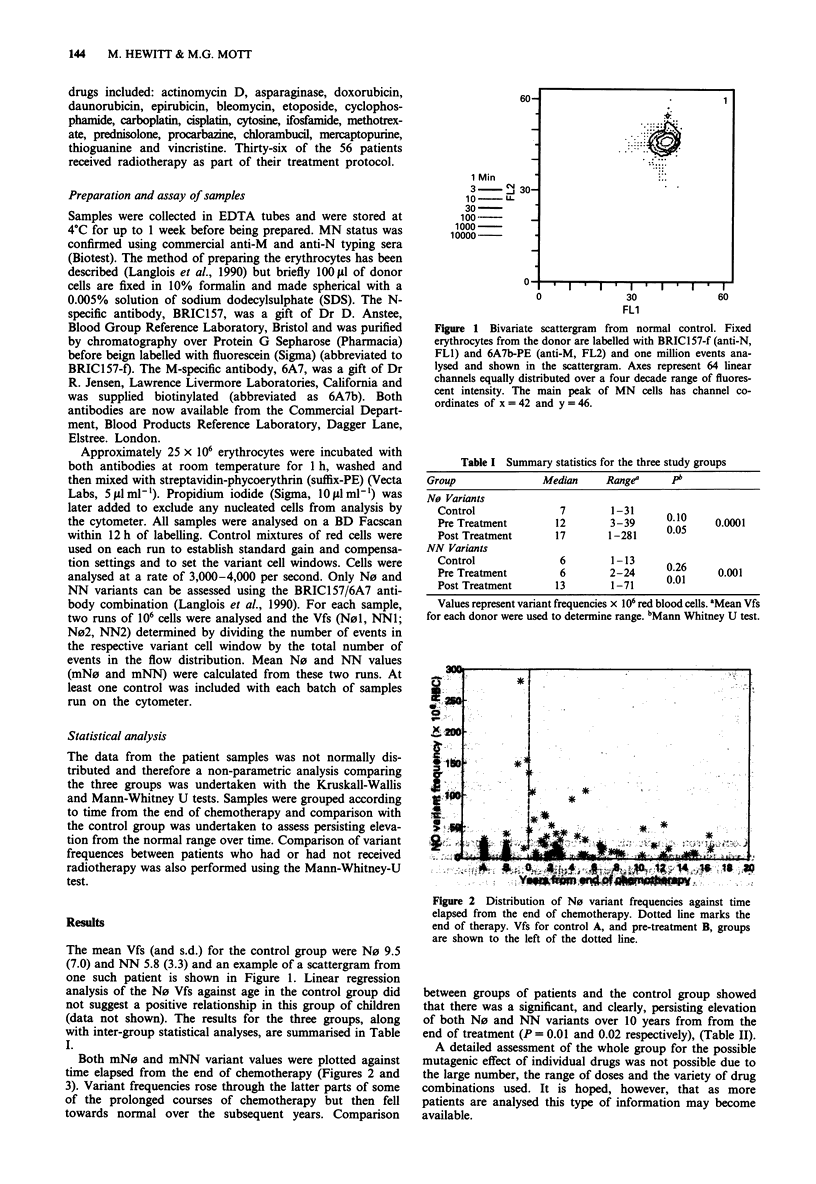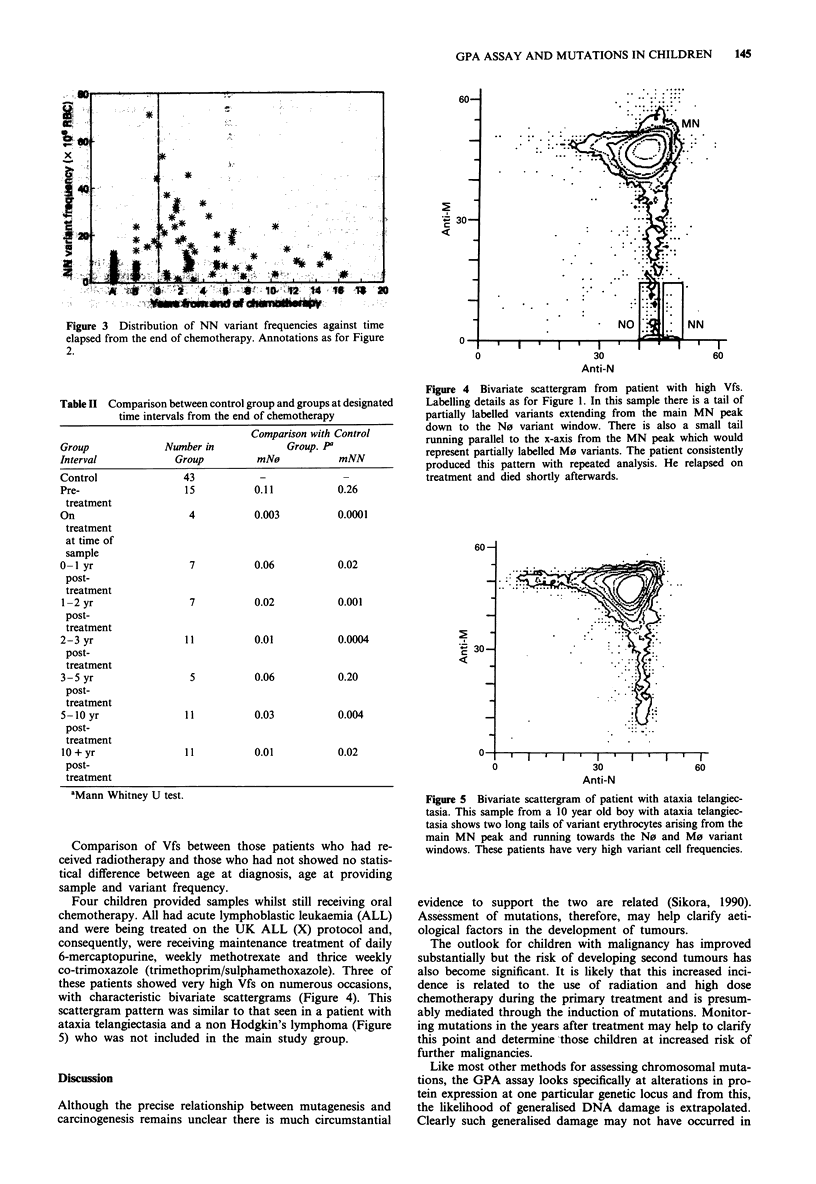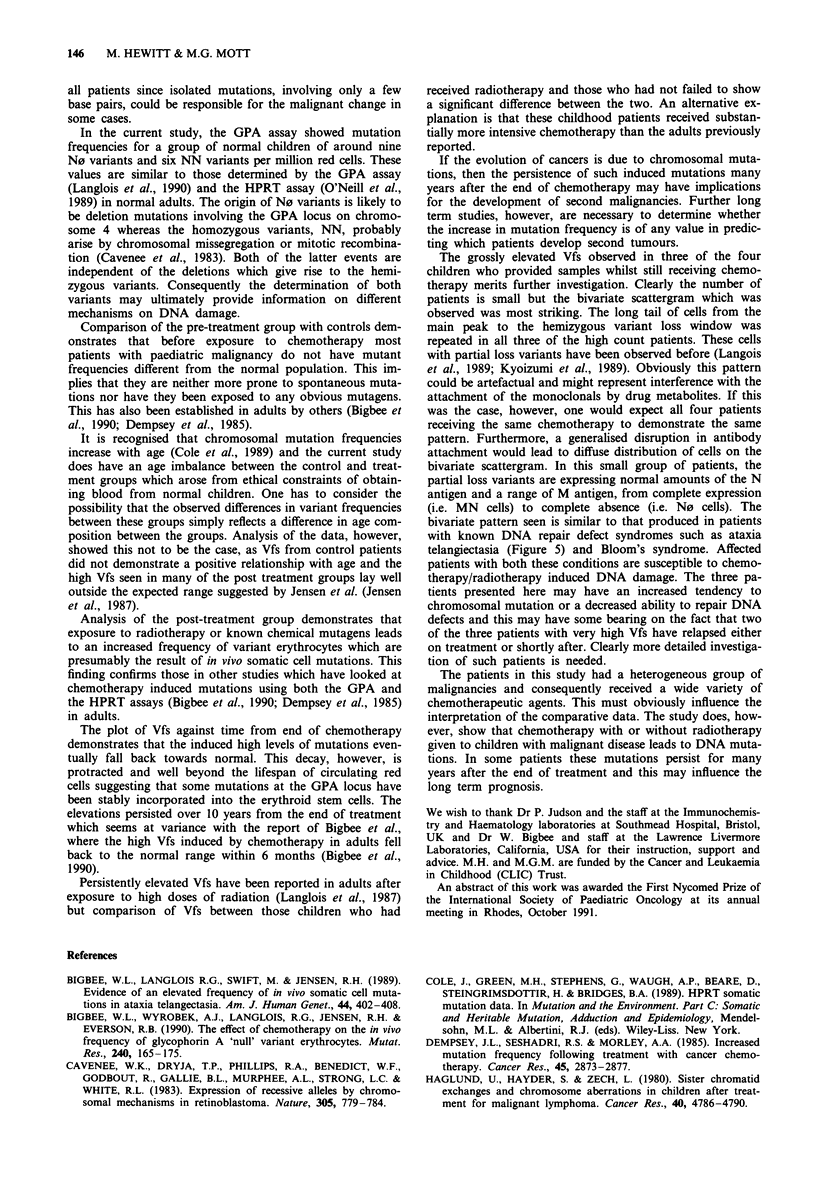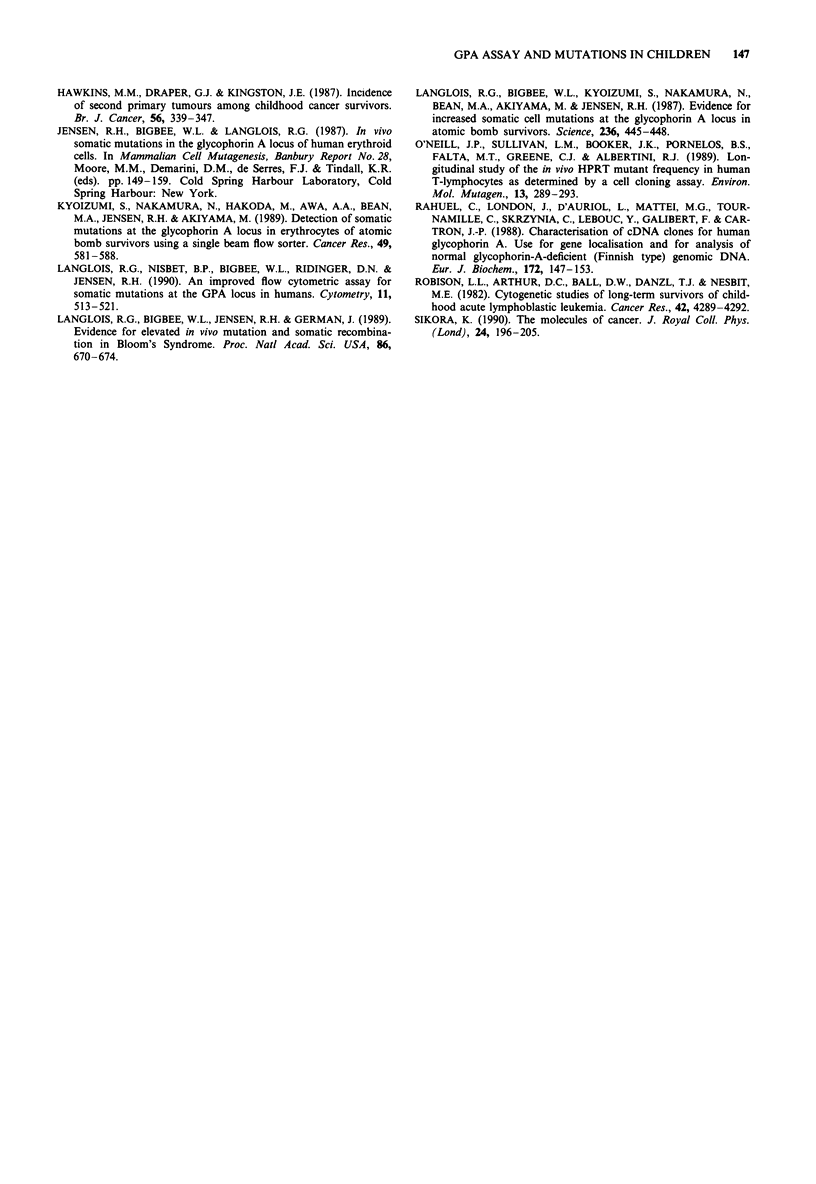# The assessment of in vivo somatic mutations in survivors of childhood malignancy.

**DOI:** 10.1038/bjc.1992.232

**Published:** 1992-07

**Authors:** M. Hewitt, M. G. Mott

**Affiliations:** Department of Paediatric Oncology, Royal Hospital for Sick Children, Bristol, UK.

## Abstract

The assessment of chromosomal mutations in children may provide information about aetiology and risk of second malignancies. A somatic cell mutation assay which determines variant erythrocytes lacking expression of an allelic form of the sialoglycoprotein, glycophorin A, was applied to samples from children before and after receiving potentially genotoxic therapy. Fifty-six children who had received treatment for their malignancy, 15 with malignancy but prior to treatment and 43 control children were assessed for the presence of Nø and NN mutant variant red cells. Control children had mean (s.d.) Nø and NN variant frequencies (Vf) of 9.5 (7.0) and 5.8 (3.3) x 10(6) erythrocytes respectively. Comparison between pre-treatment and control groups demonstrated that prior to chemotherapy, patients with paediatric malignancy do not have mutant frequencies significantly different from the normal population. Children who had received chemotherapy, with or without radiotherapy, showed a significant elevation of both Nø and NN variants over 10 years from the end of treatment. Exposure of children to radiotherapy or known chemical mutagens leads to an increased frequency of variant erythrocytes which is probably the result of in vivo somatic cell mutations. The long term implications have yet to be determined.


Br. J. Cancer (1992), 66, 143–147 © Macmillan Press Ltd., 1992

### The assessment of *in vivo* somatic mutations in survivors of childhood malignancy

M. Hewitt & M.G. Mott

*Department of Paediatric Oncology, Royal Hospital for Sick Children, Bristol, BS2 8BJ, UK.*

**Summary** The assessment of chromosomal mutations in children may provide information about aetiology and risk of second malignancies. A somatic cell mutation assay which determines variant erythrocytes lacking expression of an allelic form of the sialoglycoprotein, glycophorin A, was applied to samples from children before and after receiving potentially genotoxic therapy. Fifty-six children who had received treatment for their malignancy, 15 with malignancy but prior to treatment and 43 control children were assessed for the presence of Nø and NN mutant variant red cells. Control children had mean (s.d.) Nø and NN variant frequencies (Vf) of 9.5 (7.0) and 5.8 (3.3) $\times 10^6$ erythrocytes respectively. Comparison between pre-treatment and control groups demonstrated that prior to chemotherapy, patients with paediatric malignancy do not have mutant frequencies significantly different from the normal population. Children who had received chemotherapy, with or without radiotherapy, showed a significant elevation of both Nø and NN variants over 10 years from the end of treatment. Exposure of children to radiotherapy or known chemical mutagens leads to an increased frequency of variant erythrocytes which is probably the result of *in vivo* somatic cell mutations. The long term implications have yet to be determined.

The improved survival of children with malignancies over the last 30 years is clearly the result of more intensive treatment schedules. Radiotherapy and many chemotherapeutic drugs, however, are known to be genotoxic and chromosomal damage in treated patients has been documented (Haglund *et al.*, 1980; Robison *et al.*, 1982). An increased risk of second malignancies in treated patients has also been reported (Hawkins *et al.*, 1987) and may be related to the increased number of mutations, although the relationship between mutagenesis and carcinogenesis remains to be clarified. Survivors of childhood malignancy have a longer life span than their adult counterparts and the lifetime risk of second malignancy is therefore greater. Assessment of chromosomal damage may help to predict those children at an increased risk of second malignancy following treatment. Current methods of measuring somatic mutations, such as the HPRT assay, require large volumes of blood (20 ml) and are therefore difficult to apply in paediatric practice. The glycoprotein A (GPA) assay requires small amounts of blood (100 µl), provides rapid results (within 36 h) and does not require immediate preparation of samples. Consequently it is ideal for assessing mutations in the paediatric population or where samples have to be transported from peripheral centres. The principles of the assay have been described previously (Langlois *et al.*, 1990) but briefly it enumerates variant red cells which have undergone phenotypic change of the M and N antigens resident on the glycoprotein molecule. Heterozygotes, who express both M and N antigens can be assessed for the absence of one or other antigen (Nø or Mø variant cells) or their reduplication (NN or MM variant cells) on some red cells. Fluorescently labelled monoclonal antibodies are used to visualise these antigens and a flow cytometer to determine the variant cell frequency (Vf). This provides an assessment of chromosomal damage in the erythroid stem cells at the GPA locus – 4q28-q31 (Rahuel *et al.*, 1988). The assay has been applied to normal adults and the mean variant frequencies established as Nø = 6.2 and NN = 8.7 per million red cells (Langlois *et al.*, 1990). Adults with a known increased risk of developing malignancy have been assessed with the GPA assay – survivors of the atomic bomb (Langlois *et al.*, 1987), individuals with Bloom's syndrome (Langlois *et al.*, 1989) and ataxia telangiectasia (Bigbee *et al.*, 1989) – and have been found to have raised Vfs. Adult patients receiving chemotherapy have also been studied (Bigbee *et al.*, 1990) and demonstrate Vfs which rise through treatment, plateau and then fall back into the normal range. The current study used the GPA assay to evaluate the genotoxic effects of radiation and chemotherapy in the paediatric age group.

Correspondence: M. Hewitt, Department of Child Health, Southampton General Hospital, Southampton, SO9 4XY, UK.
Received 5 November 1991; and in revised form 31 March 1992.

### Materials and methods

### *Patients and samples*

Samples of blood for both control and study groups were obtained from children attending the Royal Hospital for Sick Children, Bristol or regionally supported clinics. Forty-three children (mean age 6.3 years; range 0.9–16.4 years) attending the hospital for routine surgery provided control samples. In 15 of the patient group, the samples were obtained before chemotherapy was started (mean age 5.4 years; range 1.6 –16.6 years) and these children are referred to as the pre-treatment group. Fifty-six patients with paediatric malignancies (mean age 13.4 years; range 2.2–32.9 years) provided samples 1 to 16.5 years after diagnosis. These children, who were aged 0.93 to 16.58 years at the time of diagnosis, formed the post-treatment group. No child in this part of the study provided more than one sample. The study was approved by the Bristol and Weston Health Authority Ethical Committee.

The criteria for selection of the patients were that they were MN phenotypes, that they had not received any blood product transfusions within the preceding 160 days and that those in the post-treatment group had received radiotherapy and chemotherapy containing at least one mutagenic drug. Children currently receiving intensive courses of chemotherapy were invariably excluded from the study as they were usually dependent upon transfused blood products. The children who had received treatment had the following maligancies (numbers in parentheses): leukaemia (22), lymphoma (eight), bone tumours (six), renal tumours (five), rhabdomyosarcomas (six), undifferentiated round cell tumours (four), neuroblastoma (three) and malignant teratoma (one). The following tumours were found in the children who formed the pre-treatment group: leukaemia (eight), bone tumours (two), renal tumours (two), rhabdomyosarcomas (one), malignant teratoma (one) astrocytoma (one) and malignant nerve sheath tumour (one). The chemotherapeutic drugs included: actinomycin D, asparaginase, doxorubicin, daunorubicin, epirubicin, bleomycin, etoposide, cyclophosphamide, carboplatin, cisplatin, cytosine, ifosfamide, methotrexate, prednisolone, procarbazine, chlorambucil, mercaptopurine, thioguanine and vincristine. Thirty-six of the 56 patients received radiotherapy as part of their treatment protocol.

### *Preparation and assay of samples*

Samples were collected in EDTA tubes and were stored at 4°C for up to 1 week before being prepared. MN status was confirmed using commercial anti-M and anti-N typing sera (Biotest). The method of preparing the erythrocytes has been described (Langlois *et al.*, 1990) but briefly 100 µl of donor cells are fixed in 10% formalin and made spherical with a 0.005% solution of sodium dodecylsulphate (SDS). The N-specific antibody, BRIC157, was a gift of Dr D. Anstee, Blood Group Reference Laboratory, Bristol and was purified by chromatography over Protein G Sepharose (Pharmacia) before beign labelled with fluorescein (Sigma) (abbreviated to BRIC157-f). The M-specific antibody, 6A7, was a gift of Dr R. Jensen, Lawrence Livermore Laboratories, California and was supplied biotinylated (abbreviated as 6A7b). Both antibodies are now available from the Commercial Department, Blood Products Reference Laboratory, Dagger Lane, Elstree. London.

Approximately $25 \times 10^6$ erythrocytes were incubated with both antibodies at room temperature for 1 h, washed and then mixed with streptavidin-phycoerythrin (suffix-PE) (Vecta Labs, 5 µl $ml^{-1}$). Propidium iodide (Sigma, 10 µl $ml^{-1}$) was later added to exclude any nucleated cells from analysis by the cytometer. All samples were analysed on a BD Facscan within 12 h of labelling. Control mixtures of red cells were used on each run to establish standard gain and compensation settings and to set the variant cell windows. Cells were analysed at a rate of 3,000–4,000 per second. Only Nø and NN variants can be assessed using the BRIC157/6A7 antibody combination (Langlois *et al.*, 1990). For each sample, two runs of $10^6$ cells were analysed and the Vfs (Nø1, NN1; Nø2, NN2) determined by dividing the number of events in the respective variant cell window by the total number of events in the flow distribution. Mean Nø and NN values (mNø and mNN) were calculated from these two runs. At least one control was included with each batch of samples run on the cytometer.

### *Statistical analysis*

The data from the patient samples was not normally distributed and therefore a non-parametric analysis comparing the three groups was undertaken with the Kruskall-Wallis and Mann-Whitney U tests. Samples were grouped according to time from the end of chemotherapy and comparison with the control group was undertaken to assess persisting elevation from the normal range over time. Comparison of variant frequences between patients who had or had not received radiotherapy was also performed using the Mann-Whitney-U test.

### Results

The mean Vfs (and s.d.) for the control group were Nø 9.5 (7.0) and NN 5.8 (3.3) and an example of a scattergram from one such patient is shown in Figure 1. Linear regression analysis of the Nø Vfs against age in the control group did not suggest a positive relationship in this group of children (data not shown). The results for the three groups, along with inter-group statistical analyses, are summarised in Table I.

Both mNø and mNN variant values were plotted against time elapsed from the end of chemotherapy (Figures 2 and 3). Variant frequencies rose through the latter parts of some of the prolonged courses of chemotherapy but then fell towards normal over the subsequent years. Comparison between groups of patients and the control group showed that there was a significant, and clearly, persisting elevation of both Nø and NN variants over 10 years from from the end of treatment ($P = 0.01$ and 0.02 respectively), (Table II).

A detailed assessment of the whole group for the possible mutagenic effect of individual drugs was not possible due to the large number, the range of doses and the variety of drug combinations used. It is hoped, however, that as more patients are analysed this type of information may become available.

**Figure 1** Bivariate scattergram from normal control. Fixed erythrocytes from the donor are labelled with BRIC157-f (anti-N, FL1) and 6A7b-PE (anti-M, FL2) and one million events analysed and shown in the scattergram. Axes represent 64 linear channels equally distributed over a four decade range of fluorescent intensity. The main peak of MN cells has channel coordinates of x = 42 and y = 46.

**Table I** Summary statistics for the three study groups

| *Group* | *Median* | *Range*[a] | *P*[b] | |
|---|---|---|---|---|
| *Nø Variants* | | | | |
| Control | 7 | 1–31 | 0.10 | |
| Pre Treatment | 12 | 3–39 | | 0.0001 |
| Post Treatment | 17 | 1–281 | 0.05 | |
| *NN Variants* | | | | |
| Control | 6 | 1–13 | 0.26 | |
| Pre Treatment | 6 | 2–24 | | 0.001 |
| Post Treatment | 13 | 1–71 | 0.01 | |

Values represent variant frequencies × $10^6$ red blood cells. [a]Mean Vfs for each donor were used to determine range. [b]Mann Whitney U test.

**Figure 2** Distribution of Nø variant frequencies against time elapsed from the end of chemotherapy. Dotted line marks the end of therapy. Vfs for control **A**, and pre-treatment **B**, groups are shown to the left of the dotted line.

**Figure 3** Distribution of NN variant frequencies against time elapsed from the end of chemotherapy. Annotations as for Figure 2.

**Table II** Comparison between control group and groups at designated time intervals from the end of chemotherapy

| *Group Interval* | *Number in Group* | *Comparison with Control Group. P*[a] *mNø* | *mNN* |
|---|---|---|---|
| Control | 43 | – | – |
| Pre-treatment | 15 | 0.11 | 0.26 |
| On treatment at time of sample | 4 | 0.003 | 0.0001 |
| 0–1 yr post-treatment | 7 | 0.06 | 0.02 |
| 1–2 yr post-treatment | 7 | 0.02 | 0.001 |
| 2–3 yr post-treatment | 11 | 0.01 | 0.0004 |
| 3–5 yr post-treatment | 5 | 0.06 | 0.20 |
| 5–10 yr post-treatment | 11 | 0.03 | 0.004 |
| 10 + yr post-treatment | 11 | 0.01 | 0.02 |

[a]Mann Whitney U test.

**Figure 4** Bivariate scattergram from patient with high Vfs. Labelling details as for Figure 1. In this sample there is a tail of partially labelled variants extending from the main MN peak down to the Nø variant window. There is also a small tail running parallel to the x-axis from the MN peak which would represent partially labelled Mø variants. The patient consistently produced this pattern with repeated analysis. He relapsed on treatment and died shortly afterwards.

**Figure 5** Bivariate scattergram of patient with ataxia telangiectasia. This sample from a 10 year old boy with ataxia telangiectasia shows two long tails of variant erythrocytes arising from the main MN peak and running towards the Nø and Mø variant windows. These patients have very high variant cell frequencies.

Comparison of Vfs between those patients who had received radiotherapy and those who had not showed no statistical difference between age at diagnosis, age at providing sample and variant frequency.

Four children provided samples whilst still receiving oral chemotherapy. All had acute lymphoblastic leukaemia (ALL) and were being treated on the UK ALL (X) protocol and, consequently, were receiving maintenance treatment of daily 6-mercaptopurine, weekly methotrexate and thrice weekly co-trimoxazole (trimethoprim/sulphamethoxazole). Three of these patients showed very high Vfs on numerous occasions, with characteristic bivariate scattergrams (Figure 4). This scattergram pattern was similar to that seen in a patient with ataxia telangiectasia and a non Hodgkin's lymphoma (Figure 5) who was not included in the main study group.

### Discussion

Although the precise relationship between mutagenesis and carcinogenesis remains unclear there is much circumstantial evidence to support the two are related (Sikora, 1990). Assessment of mutations, therefore, may help clarify aetiological factors in the development of tumours.

The outlook for children with malignancy has improved substantially but the risk of developing second tumours has also become significant. It is likely that this increased incidence is related to the use of radiation and high dose chemotherapy during the primary treatment and is presumably mediated through the induction of mutations. Monitoring mutations in the years after treatment may help to clarify this point and determine those children at increased risk of further malignancies.

Like most other methods for assessing chromosomal mutations, the GPA assay looks specifically at alterations in protein expression at one particular genetic locus and from this, the likelihood of generalised DNA damage is extrapolated. Clearly such generalised damage may not have occurred in all patients since isolated mutations, involving only a few base pairs, could be responsible for the malignant change in some cases.

In the current study, the GPA assay showed mutation frequencies for a group of normal children of around nine Nø variants and six NN variants per million red cells. These values are similar to those determined by the GPA assay (Langlois *et al.*, 1990) and the HPRT assay (O'Neill *et al.*, 1989) in normal adults. The origin of Nø variants is likely to be deletion mutations involving the GPA locus on chromosome 4 whereas the homozygous variants, NN, probably arise by chromosomal missegregation or mitotic recombination (Cavenee *et al.*, 1983). Both of the latter events are independent of the deletions which give rise to the hemizygous variants. Consequently the determination of both variants may ultimately provide information on different mechanisms on DNA damage.

Comparison of the pre-treatment group with controls demonstrates that before exposure to chemotherapy most patients with paediatric malignancy do not have mutant frequencies different from the normal population. This implies that they are neither more prone to spontaneous mutations nor have they been exposed to any obvious mutagens. This has also been established in adults by others (Bigbee *et al.*, 1990; Dempsey *et al.*, 1985).

It is recognised that chromosomal mutation frequencies increase with age (Cole *et al.*, 1989) and the current study does have an age imbalance between the control and treatment groups which arose from ethical constraints of obtaining blood from normal children. One has to consider the possibility that the observed differences in variant frequencies between these groups simply reflects a difference in age composition between the groups. Analysis of the data, however, showed this not to be the case, as Vfs from control patients did not demonstrate a positive relationship with age and the high Vfs seen in many of the post treatment groups lay well outside the expected range suggested by Jensen *et al.* (Jensen *et al.*, 1987).

Analysis of the post-treatment group demonstrates that exposure to radiotherapy or known chemical mutagens leads to an increased frequency of variant erythrocytes which are presumably the result of *in vivo* somatic cell mutations. This finding confirms those in other studies which have looked at chemotherapy induced mutations using both the GPA and the HPRT assays (Bigbee *et al.*, 1990; Dempsey *et al.*, 1985) in adults.

The plot of Vfs against time from end of chemotherapy demonstrates that the induced high levels of mutations eventually fall back towards normal. This decay, however, is protracted and well beyond the lifespan of circulating red cells suggesting that some mutations at the GPA locus have been stably incorporated into the erythroid stem cells. The elevations persisted over 10 years from the end of treatment which seems at variance with the report of Bigbee *et al.*, where the high Vfs induced by chemotherapy in adults fell back to the normal range within 6 months (Bigbee *et al.*, 1990).

Persistently elevated Vfs have been reported in adults after exposure to high doses of radiation (Langlois *et al.*, 1987) but comparison of Vfs between those children who had received radiotherapy and those who had not failed to show a significant difference between the two. An alternative explanation is that these childhood patients received substantially more intensive chemotherapy than the adults previously reported.

If the evolution of cancers is due to chromosomal mutations, then the persistence of such induced mutations many years after the end of chemotherapy may have implications for the development of second malignancies. Further long term studies, however, are necessary to determine whether the increase in mutation frequency is of any value in predicting which patients develop second tumours.

The grossly elevated Vfs observed in three of the four children who provided samples whilst still receiving chemotherapy merits further investigation. Clearly the number of patients is small but the bivariate scattergram which was observed was most striking. The long tail of cells from the main peak to the hemizygous variant loss window was repeated in all three of the high count patients. These cells with partial loss variants have been observed before (Langois *et al.*, 1989; Kyoizumi *et al.*, 1989). Obviously this pattern could be artefactual and might represent interference with the attachment of the monoclonals by drug metabolites. If this was the case, however, one would expect all four patients receiving the same chemotherapy to demonstrate the same pattern. Furthermore, a generalised disruption in antibody attachment would lead to diffuse distribution of cells on the bivariate scattergram. In this small group of patients, the partial loss variants are expressing normal amounts of the N antigen and a range of M antigen, from complete expression (i.e. MN cells) to complete absence (i.e. Nø cells). The bivariate pattern seen is similar to that produced in patients with known DNA repair defect syndromes such as ataxia telangiectasia (Figure 5) and Bloom's syndrome. Affected patients with both these conditions are susceptible to chemotherapy/radiotherapy induced DNA damage. The three patients presented here may have an increased tendency to chromosomal mutation or a decreased ability to repair DNA defects and this may have some bearing on the fact that two of the three patients with very high Vfs have relapsed either on treatment or shortly after. Clearly more detailed investigation of such patients is needed.

The patients in this study had a heterogeneous group of malignancies and consequently received a wide variety of chemotherapeutic agents. This must obviously influence the interpretation of the comparative data. The study does, however, show that chemotherapy with or without radiotherapy given to children with malignant disease leads to DNA mutations. In some patients these mutations persist for many years after the end of treatment and this may influence the long term prognosis.

We wish to thank Dr P. Judson and the staff at the Immunochemistry and Haematology laboratories at Southmead Hospital, Bristol, UK and Dr W. Bigbee and staff at the Lawrence Livermore Laboratories, California, USA for their instruction, support and advice. M.H. and M.G.M. are funded by the Cancer and Leukaemia in Childhood (CLIC) Trust.

An abstract of this work was awarded the First Nycomed Prize of the International Society of Paediatric Oncology at its annual meeting in Rhodes, October 1991.

### References

BIGBEE, W.L., LANGLOIS R.G., SWIFT, M. & JENSEN, R.H. (1989). Evidence of an elevated frequency of *in vivo* somatic cell mutations in ataxia telangectasia. *Am. J. Human Genet.*, **44**, 402–408.

BIGBEE, W.L., WYROBEK, A.J., LANGLOIS, R.G., JENSEN, R.H. & EVERSON, R.B. (1990). The effect of chemotherapy on the *in vivo* frequency of glycophorin A 'null' variant erythrocytes. *Mutat. Res.*, **240**, 165–175.

CAVENEE, W.K., DRYJA, T.P., PHILLIPS, R.A., BENEDICT, W.F., GODBOUT, R., GALLIE, B.L., MURPHEE, A.L., STRONG, L.C. & WHITE, R.L. (1983). Expression of recessive alleles by chromosomal mechanisms in retinoblastoma. *Nature*, **305**, 779–784.

COLE, J., GREEN, M.H., STEPHENS, G., WAUGH, A.P., BEARE, D., STEINGRIMSDOTTIR, H. & BRIDGES, B.A. (1989). HPRT somatic mutation data. In *Mutation and the Environment. Part C: Somatic and Heritable Mutation, Adduction and Epidemiology*, Mendelsohn, M.L. & Albertini, R.J. (eds). Wiley-Liss. New York.

DEMPSEY, J.L., SESHADRI, R.S. & MORLEY, A.A. (1985). Increased mutation frequency following treatment with cancer chemotherapy. *Cancer Res.*, **45**, 2873–2877.

HAGLUND, U., HAYDER, S. & ZECH, L. (1980). Sister chromatid exchanges and chromosome aberrations in children after treatment for malignant lymphoma. *Cancer Res.*, **40**, 4786–4790.

HAWKINS, M.M., DRAPER, G.J. & KINGSTON, J.E. (1987). Incidence of second primary tumours among childhood cancer survivors. *Br. J. Cancer*, **56**, 339–347.

JENSEN, R.H., BIGBEE, W.L. & LANGLOIS, R.G. (1987). *In vivo* somatic mutations in the glycophorin A locus of human erythroid cells. In *Mammalian Cell Mutagenesis, Banbury Report No. 28*, Moore, M.M., Demarini, D.M., de Serres, F.J. & Tindall, K.R. (eds). pp. 149–159. Cold Spring Harbour Laboratory, Cold Spring Harbour: New York.

KYOIZUMI, S., NAKAMURA, N., HAKODA, M., AWA, A.A., BEAN, M.A., JENSEN, R.H. & AKIYAMA, M. (1989). Detection of somatic mutations at the glycophorin A locus in erythrocytes of atomic bomb survivors using a single beam flow sorter. *Cancer Res.*, **49**, 581–588.

LANGLOIS, R.G., NISBET, B.P., BIGBEE, W.L., RIDINGER, D.N. & JENSEN, R.H. (1990). An improved flow cytometric assay for somatic mutations at the GPA locus in humans. *Cytometry*, **11**, 513–521.

LANGLOIS, R.G., BIGBEE, W.L., JENSEN, R.H. & GERMAN, J. (1989). Evidence for elevated *in vivo* mutation and somatic recombination in Bloom's Syndrome. *Proc. Natl Acad. Sci. USA*, **86**, 670–674.

LANGLOIS, R.G., BIGBEE, W.L., KYOIZUMI, S., NAKAMURA, N., BEAN, M.A., AKIYAMA, M. & JENSEN, R.H. (1987). Evidence for increased somatic cell mutations at the glycophorin A locus in atomic bomb survivors. *Science*, **236**, 445–448.

O'NEILL, J.P., SULLIVAN, L.M., BOOKER, J.K., PORNELOS, B.S., FALTA, M.T., GREENE, C.J. & ALBERTINI, R.J. (1989). Longitudinal study of the *in vivo* HPRT mutant frequency in human T-lymphocytes as determined by a cell cloning assay. *Environ. Mol. Mutagen.*, **13**, 289–293.

RAHUEL, C., LONDON, J., D'AURIOL, L., MATTEI, M.G., TOURNAMILLE, C., SKRZYNIA, C., LEBOUC, Y., GALIBERT, F. & CARTRON, J.-P. (1988). Characterisation of cDNA clones for human glycophorin A. Use for gene localisation and for analysis of normal glycophorin-A-deficient (Finnish type) genomic DNA. *Eur. J. Biochem.*, **172**, 147–153.

ROBISON, L.L., ARTHUR, D.C., BALL, D.W., DANZL, T.J. & NESBIT, M.E. (1982). Cytogenetic studies of long-term survivors of childhood acute lymphoblastic leukemia. *Cancer Res.*, **42**, 4289–4292.

SIKORA, K. (1990). The molecules of cancer. *J. Royal Coll. Phys. (Lond)*, **24**, 196–205.